# High-Resolution Geospatial Analysis of Dengue Vulnerability in Urban and Rural Areas of San Luis Potosí, Mexico

**DOI:** 10.3390/tropicalmed10110307

**Published:** 2025-10-28

**Authors:** Darío Gaytán Hernández, Daniel Sánchez Hernández, Luis Eduardo Hernández Ibarra, Enrique Ibarra Zapata, Omar Parra Rodríguez, Verónica Gallegos García, Omar Medina de la Cruz, Marisol Gallegos García

**Affiliations:** 1 Faculty of Nursing and Nutrition, Autonomous University of San Luis Potosí, Av. Niño Artillero #130, Zona Universitaria, San Luis Potosí 78240, SLP, Mexico; 2 Faculty of Social Sciences and Humanities, Autonomous University of San Luis Potosí, Av. Industrias #101-A, Workshop Subdivision, San Luis Potosí 78240, SLP, Mexico; 3 Faculty of Agronomy, Autonomous University of San Luis Potosí, Km. 14.5 Carretera San Luis Potosí, Matehuala, Ejido Palma de la Cruz, Soledad de Graciano Sánchez 78321, SLP, Mexico; 4 Environmental Agenda, Universidad Autónoma de San Luis Potosí, Dr. Manuel Nava #221, Zona Universitaria, San Luis Potosí 78397, SLP, Mexico; 5 Faculty of Engineering, Autonomous University of San Luis Potosí, Av. Dr. Manuel Nava #304, Zona Universitaria, San Luis Potosí 78240, SLP, Mexico

**Keywords:** dengue virus, receiver operating characteristic curve, spatial analysis, geographic information system, risk factors

## Abstract

Objective: The aim was to analyze the temporal evolution and spatial distribution of classic and hemorrhagic dengue in the Mexican state of San Luis Potosí at the basic geostatistical area (BGA) level and to develop multivariate models to estimate the population’s degree of vulnerability. Methodology: Classic and hemorrhagic dengue cases for 2015–2020 were obtained from the Mexican Ministry of Health, georeferenced at the pixel level, and subsequently grouped by BGA. Environmental, proximity, and social variables were obtained from official sites: IMTA, SMN, USGS, and INEGI. Multivariate logistic regression models were developed using PASW Statistics v. 18 software to estimate the degree of vulnerability, and the receiver operating characteristic curve was used to validate them. Results: A total of 125, 128, 109, 624, 1580, and 1817 dengue cases were identified for 2015, 2016, 2017, 2018, 2019, and 2020, respectively. The major factors contributing to the vulnerability of classic dengue fever included population, temperature, and distance to agricultural areas. For hemorrhagic dengue, the contributing factors were temperature, population, and mean annual rainfall. Vulnerability prediction was determined by taking the area under the curve values, which were 0.957 for classic dengue fever and 0.930 for hemorrhagic dengue, both indicating a “very good ability” to predict. Conclusions: These results can be used to design and implement targeted strategies, particularly for modifiable factors, such as prevention measures directed towards populated areas and the improvement of sewage systems, in addition to non-modifiable factors, such as temperature and rainfall. This method can be replicated as an additional tool to address this public health issue.

## 1. Introduction

Dengue is an infectious viral disease caused by the dengue virus, of which there are four serotypes, and is spread through mosquito bites [1]. Consequently, individuals can be infected up to four times. The primary vectors for this disease are *Aedes aegypti* mosquitoes, with *Aedes albopictus* playing a secondary role in transmission.

Dengue is a global public health problem; in 2024, the World Health Organization (WHO) estimated that half of the world’s population is at risk of contracting dengue, with between 100 and 400 million infections occurring annually. Although most people who contract dengue are asymptomatic, the disease can sometimes worsen, requiring hospitalization; in severe cases, it may be fatal [2]. Moreover, in 2023, it was reported that there were 2.8 million recorded cases of dengue in the Americas in the year 2022, more than double the 1.2 million cases reported in 2021 [1].

In Mexico, the Secretary of Health in epidemiological week 52 of 2022 revealed that 6746 cases of dengue were reported in 2021; this number nearly doubled in 2022, reaching 12,671 cases [3]. Similarly, in epidemiological week 52 of 2023 in the Mexican state of San Luis Potosí, it was revealed that 566 confirmed cases were reported in 2023, with 108 cases exhibiting severe to alarming symptoms [4].

Dengue primarily occurs in tropical and subtropical regions worldwide, particularly in urban and semi-urban areas [2]. However, Man et al. (2023) conducted a systematic search for articles that evaluated the prevalence or cumulative incidence of dengue and found that its incidence in rural areas can equal or exceed that in urban regions [5]. To add to this problem, in 2023, the United Nations Organization (UNO) declared that global warming is expected to increase dengue cases worldwide [6]. In a study carried out in Colombia, in three different ecosystems, using generalized linear models and generalized aditivis models to examine geographic data. The results showed that various factors such as migratory movements, inadequate sanitation, inappropriate water supply, among others, favor the development and spread of the vector [7].

Relevant studies on dengue have used spatial and statistical modeling. such as the one developed to predict the geographical distribution of dengue cases in the metropolitan region of São Paulo, Brasil; MaxEnt distribution modeling was applied, incorporating sociodemographic and housing data [8]. Recently, several high-resolution geospatial approaches have advanced the analysis of dengue risk. A study was done in an Amazon sub region of Colombia to develop dengue risk maps, based on the ordinary least squares regression technique and multicriteria analysis [9]. Another investigation in Bhopal, India, combining Geographic Information Systems with machine-learning models to delineate risk areas from environmental, demographic, and infrastructural variables, demonstrating progress toward more precise predictive frameworks [10]. In research conducted in Zhongshan, China, producing one-kilometer-scale risk maps that captured fine spatial variability in regions with limited entomological data [11].

In Dhaka, Bangladesh, a study was developed, applying a multicriteria decision-making approach (MCDM + GIS) that integrated weighted environmental and demographic factors to estimate dengue susceptibility in densely populated urban settings. Collectively, these studies illustrate the consolidation of geospatial methodologies grounded in multivariable analysis and fine spatial resolution, designed to enhance the precision of vulnerability assessment and to clarify the influence of socio-environmental factors on dengue distribution [12].

Dengue became endemic in Mexico during the 1980s, prompting governmental initiatives aimed at prevention and control through program implementation. However, these efforts were insufficient to curb the rise in cases, which spread to over 90% of the country’s states by 2000 [13]. Dengue cases have increased significantly throughout the state of San Luis Potosí, even in municipalities where this disease did not occur, so it is important to know the conditions that favor the development and spread of the Aedes aegypti vector. Consequently, this study aims to develop geospatial and statistical models to estimate vulnerability to classic dengue and hemorrhagic dengue fever at the rural and urban basic geostatistical area (BGA) level in San Luis Potosí State, Mexico. It also seeks to assess these temporal evolution and spatial distribution of these dengue variants at the BGA level, advancing multivariate models to determine population vulnerability.

## 2. Materials and Methods

### 2.1. Study Area

**Figure 1:**
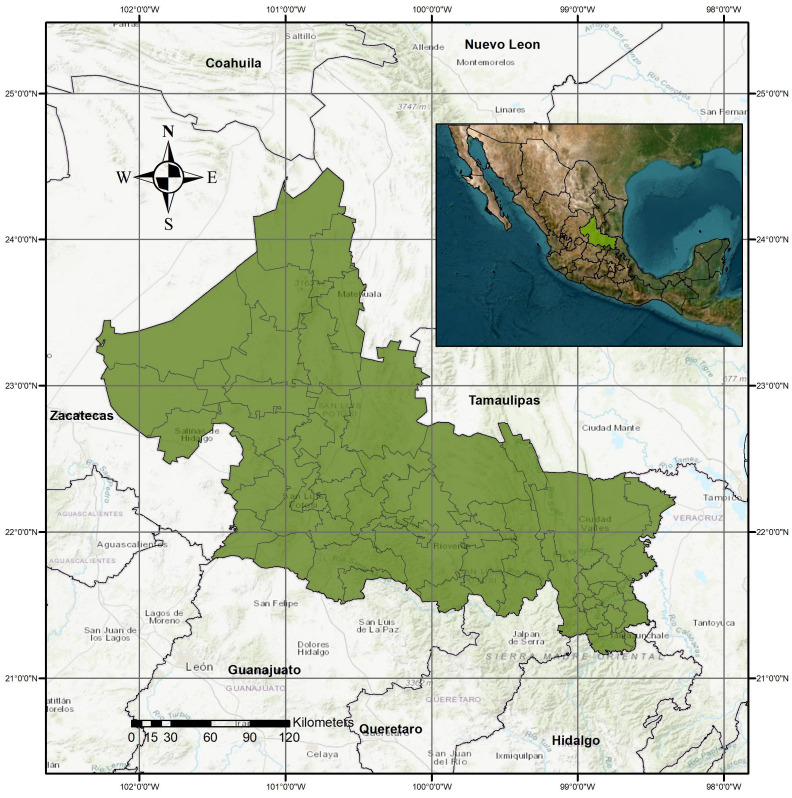
Location of the study area.

San Luis Potosí, located in northeastern–central Mexico, is one of the most biodiverse states in the country, covering approximately 61,137 km^2^ and comprising 58 municipalities [14], as illustrated in Figure 1.

### 2.2. Available Data

Data were obtained from the Department of Health of the San Luis Potosí State Government [15], encompassing all cases of dengue that occurred between 2015 and 2020, comprising 4180 cases of classic dengue fever and 203 hemorrhagic dengue cases. A total of 4383 dengue cases were georeferenced using the Universal Transverse Mercator (UTM) projection-zone 14. Precise geographic coordinates of each dengue case were obtained using Google Earth, Google Maps, Heraldo Maps, Roadmap, Satellite-Maps, Map-Carta, and postal code databases (Código.mx).

### 2.3. Variables

Four factors (environmental, proximity, location, and social) were considered as independent variables contributing to the abundance of mosquitoes. All values were obtained at the pixel level (30 m) across rural and urban areas and then grouped by rural and urban BGA level. Environmental Variables: Elevations of all municipalities in San Luis Potosí were obtained using the INEGI Digital Elevation Model [16]. Temperature and rainfall data were gathered from all 83 weather stations in the state [17,18]. These values were interpolated using the inverse distance weighted algorithm to achieve their pixel-level values through rasterization. Humidity values were derived from the Climate Research Unit’s historical archives and interpolated using the ordinary Kriging method [19,20,21].

For proximity variables, the Euclidean distance algorithm was employed to determine proximity values to the nearest body of water, wooded areas, grasslands, agricultural areas, and roads or paths by calculating the metric distance from each pixel in the raster to its nearest location [22,23].

For land use mapping, a supervised classification using the maximum likelihood algorithm was performed on Landsat spectral data to extract bodies of water and wooded areas [24]. Landsat 8 Operational Land Imager images were downloaded from Glovis for scenes 028-043, 028-044, 028-045, and 029-044, with dates ranging from 16 November 2021 to 25 December 2021 [25]. These products underwent systematic geometric corrections, utilizing ground control points or shipboard position information to deliver images recorded in a map projection referenced to WGS84, G873, or its current version. Training fields were digitized from land use and land cover features over the imagery and supported by a land use/land cover map [23,26]. Following the Landsat-based spectral data classification, validation was conducted by constructing an error matrix using field control point data, achieving an overall accuracy of 95.38% and a Kappa of 0.91 [27].

**Figure 2:**
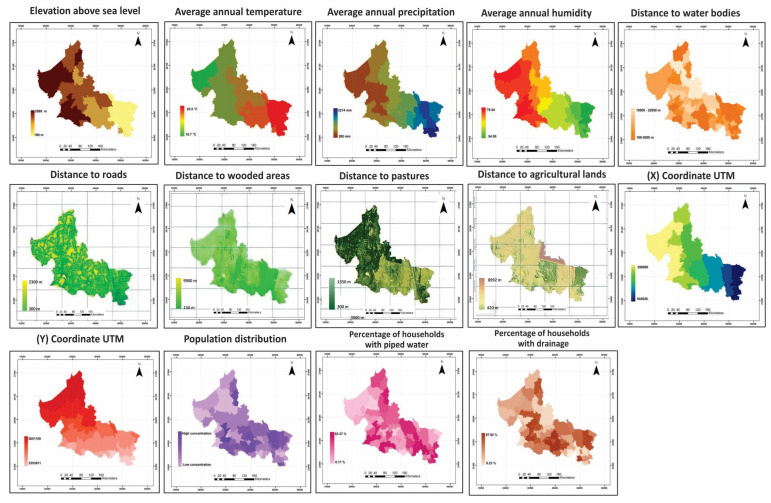
Variables used and study area state context.

Location Variables: To identify spatial trends in dengue outbreaks, the X and Y UTM coordinates (XUTM, YUTM) were used [28]. Horizontal (XUTM) and vertical (YUTM) sweeps were performed over the study area utilizing IDRISI Selva v.17.0 software, generating two raster-format files: XUTM and YUTM (Figure 2).

Social Variables: The population size affects the probability of contracting dengue [29]. Local population data were sourced from the 2020 Geostatistical Framework and interpolated using the ordinary Kriging method [30]. To explore social inequalities and their relationship to dengue vulnerability [31], data were gathered on two social variables indicating living conditions: the percentage of homes with piped water, and those with drainage systems [32]. These indices were obtained by rasterizing municipal boundaries, using the index of interest as a pixel value (Table 1).

All variables included in the analysis were rasterized considering the extreme coordinates.

**Figure 3:**
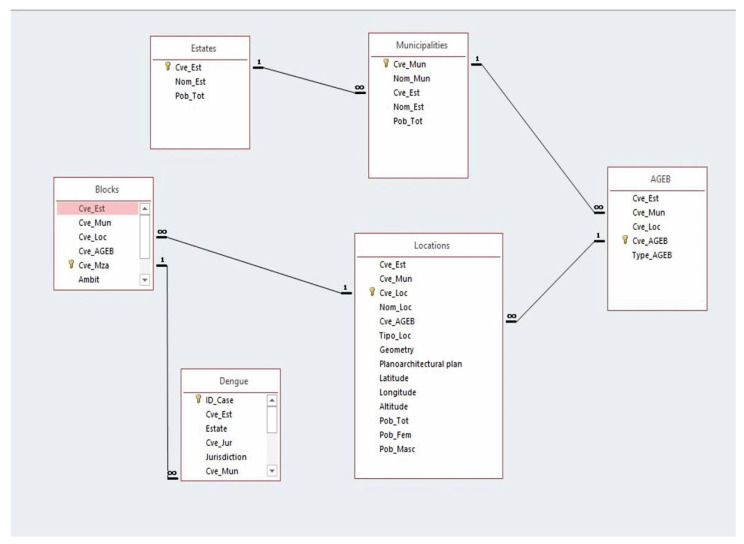
Entity–relationship model of the dengue case database 2015–2020.

A database was constructed in Microsoft Access to develop the entity–relationship model, which includes all the tables that form the structure and are related through their primary keys, establishing a 1-to-many relationship. This model enabled the creation of a foreign key named the locality key, which is located within the table labeled ‘dengue.’ This table encompasses all the cases from the period of 2015–2020, and through this key was linked to the table containing all rural and urban localities of San Luis Potosi. Consequently, the coordinates for each dengue case were obtained (Figure 3).

A spatial resolution grid of 1000 m by 1000 m was constructed as a spatial reference; however, the spatial analysis was carried out at the BGA level using the official polygons in INEGI shp format, classifying 7545 pixels. Within these pixels, the coordinates of 4180 cases of classic dengue fever and 203 cases of dengue hemorrhagic fever were located, leaving 3162 pixels without registered dengue cases. These were subsequently grouped by BGA. To assess vulnerability to dengue at the BGA level, which may be influenced by each independent variable individually, the 7545 spatial units and all the dengue cases registered during the studied period were used.

The software Access, Excel, ArcMap 10.1, and PASW Statistics v. 18 were employed to estimate and compare the means, using one-way OAOV, of the values of the independent variables among BGAs without dengue, BGAs with dengue hemorrhagic fever, and BGAs with classic dengue fever. A binary logistic regression was conducted; this statistical tool is appropriate when the dependent variable can have only two values: 1 when the condition is present and 0 when it is absent [34], with dengue cases as the dependent variable. The result in each model is expressed in terms of probability, ranging from 0 to 1, where 1 indicates the presence of dengue and 0 its absence (probability > 0.5 for presence, and 0 for probability < 0.5) [35].

Two multivariate binary logistic regression models were also developed, one for cases of classic dengue fever and the other for hemorrhagic dengue, to estimate dengue vulnerability at the BGA level, which may result from the collective influence of independent variables, the beta coefficients are given in natural logarithm, to convert them to original units, the exponential is applied. The final models were refined by excluding independent variables exhibiting multicollinearity [34].

Multiple linear regression was utilized to validate the absence of multicollinearity [36], both for classic and hemorrhagic dengue, considering a variance inflation factor ≤ 10 as indicative of no multicollinearity [36]. The discriminative ability of the multivariate models was evaluated using the receiver operating characteristic curve. This statistical method is useful in determining the diagnostic accuracy of the models to estimate the probability of detecting true positives (sensitivity) versus false positives (1-specificity) [37].

The area under the receiver operating characteristic curve is a measure that quantifies the goodness of fit for the models and was used as a potential predictive indicator, with values ranging between 0.5 and 1 [37]. Additionally, the optimal cut-off point was identified (i.e., the value with the highest sensitivity and the lowest number of false positives [1 = specificity]), for which the Youden index was employed [38]. The Kappa coefficient was applied to evaluate the concordance between recorded dengue cases and those estimated by the multivariate models [27].

The independent variables were transformed to a standardized scale to be comparable before performing the logistic regression [39]. The following expression was used:

$Z=\frac{\left(x-\;¯\;x\right)}{S}$, where *Z* and x are the transformed and original explanatory variables, respectively,  ¯x is the arithmetic mean, and *S* is the standard deviation. This approach facilitates identifying each variable’s impact on dengue vulnerability and aids in interpretation. To estimate dengue levels of risk and the percentage of the San Luis Potosi population potentially affected at the BGA level, the following five-level risk scale was used: very low (0–0.199), low (0.2–0.399), medium (0.4–0.599), high (0.6–0.799), and very high (0.8–1) [40].

## 3. Results

**Figure 4:**
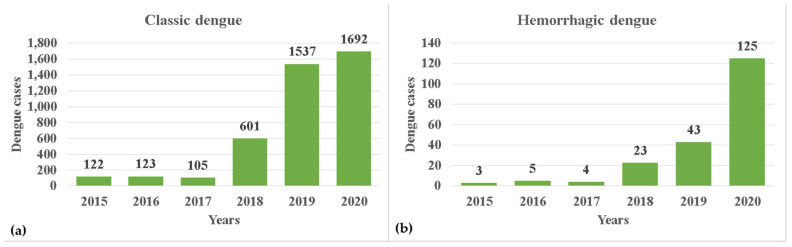
Temporal evolution of dengue cases (2015–2020) in the Mexican state of San Luis Potosi. (**a**) Classic dengue (**b**) Hemorrhagic dengue.

During the study period, 4383 cases of dengue were recorded. The evolution of classic dengue increased each year during the study period, except in 2017, the cumulative number reached 4180 (Figure 4a). Meanwhile, the evolution of hemorrhagic dengue showed constant growth each year, reaching a cumulative total of 203. This means that for every case reported, 20.59 cases of classic dengue were reported (Figure 4b).

Regarding the independent variables, more than 50% have a standard deviation greater than their respective means. Considering the mean and median, one can observe that, in the absence of dengue, the values are lower for the following variables: average annual temperature, average annual rainfall, and population. Conversely, the values are higher for the percentage of homes with piped water and those with drainage. For the remaining variables, a priori, it is not possible to specify any others. The reality is that the combination of these variable values can favor mosquito development (Table 2).

### 3.1. Bivariate Logistic Regression Analysis

All independent variables (factors) demonstrated a statistically significant relationship with the two types of dengue, except for the variable ‘Elevation above sea level’ in hemorrhagic dengue. Aside from ‘Elevation above sea level,’ the beta coefficients for all environmental variables were positive, indicating that they are risk factors. In contrast, variables related to proximity had negative coefficients, suggesting that an increase in these variables leads to a decrease in risk. This is similarly observed for the percentage of homes with piped water and homes with drainage. Conversely, the variable ‘Population’ has a positive coefficient, indicating that as the population increases, so does the risk (Table 3).

### 3.2. Preliminary Multivariate Logistic Regression Analysis

Table 4 reveals that the variable distance to agricultural areas was not statistically significant, whereas eight variables were not significant for dengue hemorrhagic fever. However, both models indicated multicollinearity among several factors, such as distance to roads for classic and hemorrhagic dengue fever.

### 3.3. Final Multivariate Logistic Regression Analysis

When variables that exhibited multicollinearity were excluded from the models, all variables were statistically significant in both types of dengue. In both models, the signs of the beta coefficients align with theoretical expectations; an increase in variables with negative beta coefficients indicates a decreased risk of contracting dengue, while those with positive coefficients increase it. Additionally, the omnibus tests for classic and hemorrhagic dengue showed *p* < 0.001, allowing us to interpret the logistic model. This suggests that at least one is significantly different from zero among all the model coefficients.

The models with standardized variables identified the same three risk factors for vulnerability to classic and hemorrhagic dengue. The most significant factor for hemorrhagic dengue was the mean annual temperature at the BGA level, whereas for classic dengue fever, it was the second most significant factor. Significant differences were observed in the means and medians, which were lower in BGAs without dengue, followed by BGAs with hemorrhagic dengue, and, finally, those with classic dengue fever. The mean and median temperatures of BGAs with dengue were 22.9 and 24.2 °C, respectively, with minimum and maximum temperatures ranging from 16.8 to 27.1 °C.

The population of the BGA is the primary factor for classic dengue fever and the third for hemorrhagic dengue. Increasing population density heightens vulnerability because an infected female mosquito is more likely to infect additional individuals. Similarly, rainfall was identified as the second most significant factor for hemorrhagic dengue and the third for classic dengue fever (Table 5).

The Kappa test result, assessing the concordance between BGAs with registered cases and those without versus the presence or absence of dengue estimated by the models for the same BGAs, showed a Kappa coefficient of 0.791 (*p* < 0.001) for classic dengue fever and a Kappa of 0.459 (*p* < 0.001) for dengue hemorrhagic fever. Over 50% of the population was identified as having a high risk (Table 6).

**Figure 5:**
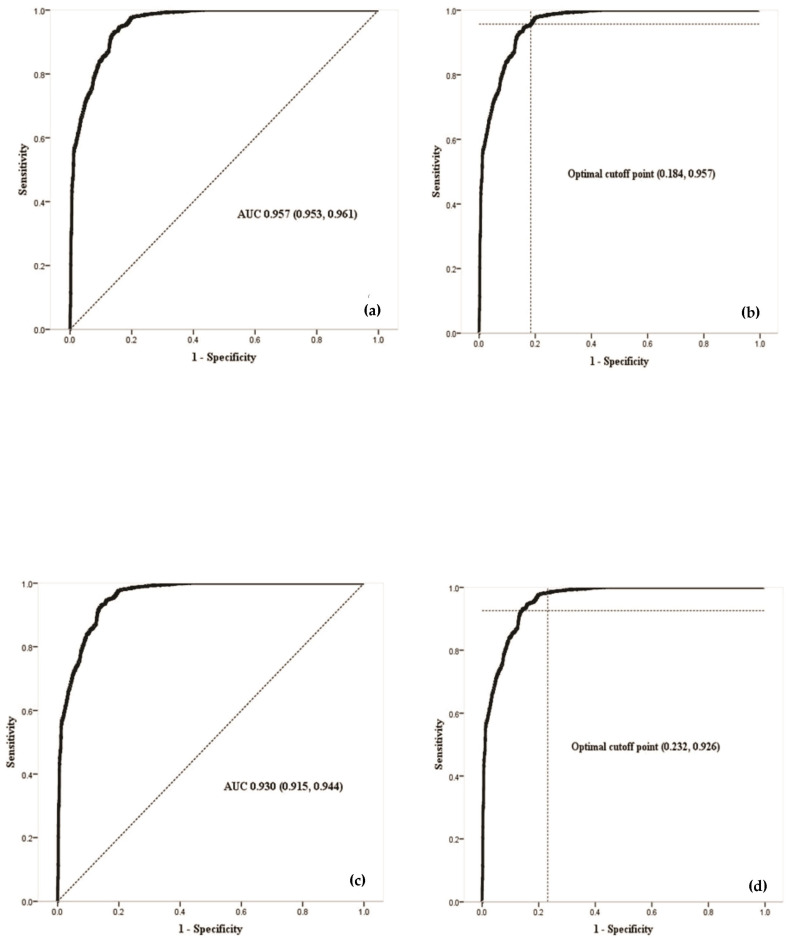
ROC curve. (**a**) Area under the curve for classic dengue fever; (**b**) optimal decision point to precision identify a true positive or true negative result for classic dengue fever; (**c**) area under the curve for hemorrhagic dengue; (**d**) optimal decision point to precision identify a true positive or true negative result for hemorrhagic dengue.

Figure 5 illustrates that the area under the curve demonstrates a strong ability to correctly distinguish whether a particular BGA has dengue, with values of 0.957 (Figure 5a) for classic dengue fever, and 0.930 (Figure 5c) for hemorrhagic dengue. In both instances, the confidence interval does not include 0.5, indicating a significant difference in the BGAs susceptible to dengue. The optimal point where sensitivity and specificity are maximized is 0.975 sensitivity and 0.816 specificity for classic dengue fever. For hemorrhagic dengue, these values are 0.926 and 0.768, respectively. Furthermore, the optimal decision threshold for BGA accurately identifying a BGA as having a true positive or true negative result was determined to be (0.184, 0.957) for classic dengue fever (Figure 5b) and (0.232, 0.926) for hemorrhagic dengue Figure 5d. At these thresholds, the maximum difference between sensitivity and 1-specificity was noted.

The dotted diagonal line, called non-discriminatory, indicates that the sensitivity and specificity values are equal to 0.5 (Figure 5a,c). The dotted lines indicate the coordinates for identifying the optimal point (where they intersect) to accurately identify a true positive or negative result (Figure 5b,c). 

**Figure 6:**
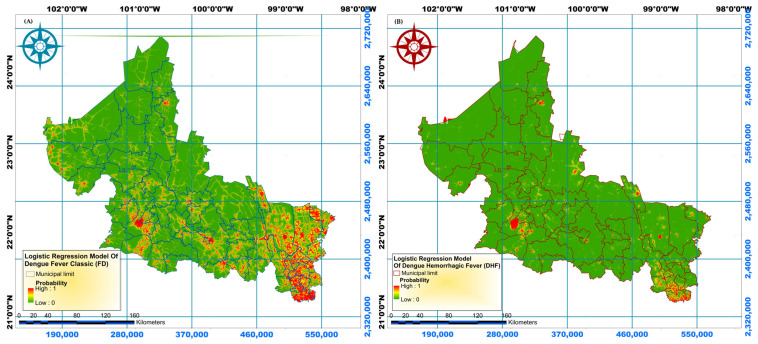
Spatial distribution of dengue fever (**A**) Classic dengue (**B**) Hemorrhagic dengue.

Classic dengue cases are located mainly in the Huasteca Zone and in the most populated municipalities such as San Luis Potosí and Soledad de Graciano Sánchez, and there are also cases in the north of the State (Figure 6A). While cases of hemorrhagic dengue are less frequent, they follow a similar pattern to those of classic dengue. However, it is clear that in the north of the state, the problem is more serious than with classic dengue (Figure 6B).

## 5. Discussion

In this study, 13.7% of classic dengue cases and 27.6% of hemorrhagic dengue cases were reported to occur in the desert and colder areas of the central and northern parts of the state, where dengue had not previously been reported. This shift is attributed to changes in infection patterns. As noted in [22], these changes are driven by extreme meteorological phenomena, climate change, and the El Niño phenomenon (it is a natural and recurring weather pattern that affects the global climate. It warms the surface of the eastern and central Pacific Ocean), in conjunction with the adaptive capacity of mosquitoes [41].

The Huasteca region features characteristics conducive to the proliferation of the Aedes aegypti mosquito, with climates ranging from warm humid and warm subhumid to temperate humid. Temperatures can fluctuate between 50 °C and −2 °C, and rainfall is abundant [42]. Several factors contributing to this health problem are interrelated, such as rainfall and humidity [1,43], as well as sanitation, drainage, and population density [2]. Therefore, multivariate logistic models developed for this study are robust in evaluating vulnerability to dengue as they consider the synergies between independent variables, where the simultaneous presence of several risk factors not only adds to vulnerability but also multiplies risk [44]. Thus, the interpretation of each factor in assessing vulnerability assumes that other factors remain constant.

The mean annual temperature and median recorded in this study are lower in the BGAs without dengue (between 0 and 9.9 °C) and with dengue (between 22.2 and 24.2 °C), indicating a relationship between this variable and the disease and is within the ranges of several studies, such as [8], which identified that mean temperatures between 18 and 25 °C create environments suitable for dengue development. Ref. [45] confirmed that optimal temperature ranges vary between 23 and 29 °C. Ref. [46] determined that thermal levels conducive to dengue propagation occur at 29 °C, whereas [47] found that the mosquito lifespan shortens at 30 °C and extends significantly at 26 °C. Ref. [48] noted that mosquito development and survival increase at 26–28 °C.

Applying the exponential to the beta coefficient of temperature (exp(0.198)), this study found that for each 1 °C increase in temperature in the BGA, and keeping the rest of the model variables fixed, the vulnerability risk increased by 21.9% for classic dengue fever; this risk was not significant for hemorrhagic dengue (Table 4). Additionally, the mean and median population values were higher in BGAs with dengue presence. Of the examined cases, 47.1% of classic dengue fever and 57.3% of hemorrhagic dengue were reported in the ten municipalities with the highest population density per km^2^ [30]. Furthermore, vulnerability to dengue increased by 0.02% for classic dengue fever and 0.007% for classic dengue fever and hemorrhagic dengue, respectively, for each additional inhabitant in the BGA, keeping the rest of the model variables fixed (Table 4). This finding is consistent with [2], which states that population growth is a risk factor for mosquito development. Ref. [49] also found that the incidence ratio of dengue increases with rising population density per km^2^. Ref. [50] supports the relationship between population density and dengue cases. Ref. [51] showed that a population density of over 1000 inhabitants per km^2^ in urban areas is associated with significant increases in dengue cases. In contrast, refs. [52,53] found no significant correlation between population density and dengue. Certain high-density urban sectors are inhabited predominantly by upper social classes, which have efficient infrastructure and heightened awareness of risk.

In this study, we found that for each millimeter increase in rainfall, and keeping the rest of the model variables fixed, the vulnerability of the BGA to classic and hemorrhagic dengue increased by 0.09% and 0.15%, respectively (Table 4). Additionally, both the mean and the median are higher in BGAs with recorded dengue cases. Ref. [54] found a positive correlation between the number of dengue cases and rainfall. Ref. [55] observed an r = 0.6214 between the magnitude of rainfall and the recurrence of dengue cases in Paraguay. Furthermore, ref. [56] found a correlation between dengue cases and rainfall ranging from 83 to 15 mm; each rainy day increased the dengue incidence rate. Refs. [49,57] confirmed that rainfall is related to the oviposition of female mosquitoes.

The mean and median humidity levels were higher in BGAs with dengue cases. Moreover, in the bivariate analysis (Table 3), humidity was identified as a risk factor for classic and hemorrhagic dengue vulnerability, consistent with [54], who found that a 1% increase in humidity corresponded to a linear increase in dengue cases. Humidity was also used as a variable factor to estimate the incidence of various diseases, including dengue [58]. Ref. [59] reported that female mosquitoes survived twice as long between 25 °C and 80% humidity. This result indirectly aligns with the present findings, as the medians of these two independent variables are close to the published values. Nevertheless, humidity emerged as a protective factor in the multivariate models (Table 4 and Table 5). This contradictory relationship could be partially explained by considering all analyzed factors’ simultaneous effects.

Land use and cover changes play a crucial role in the spread of vector-borne diseases. The results indicate that the farther a BGA is from agricultural areas, the lower the risk for vulnerability to classic dengue fever. The mean is higher in BGAs with dengue cases. These findings are consistent with those of [60], who confirmed the recurrence of dengue cases close to agricultural areas. This study found that individuals living one meter farther from agricultural areas were 0.04% less likely to contract classic dengue and 0.05% less likely to contract hemorrhagic dengue (Table 3), similar to the findings of [19], who located vector breeding sites in plantations in Sri Lanka. Ref. [61] concluded that farmers near plantations have an almost eightfold increased risk of dengue virus infection (relative risk 7.94, 95% CI 2.29–27.5).

Ref. [62] argued that the global expansion of agriculture intensifies the spread of vector-borne diseases because vegetation and bodies of water provide ideal habitats for mosquitoes. Additionally, fumigations eliminate mosquitoes’ natural predators, leading to an overpopulation of vectors, including *Aedes aegypti* [63]. The median elevation above sea level is lower in the BGAs where dengue was not present. However, elevation proved to be a protective factor against vulnerability in the final multivariate model for classic dengue fever, although no general consensus exists regarding the altitude at which Aedes aegypti mosquitoes can survive. According to [64], elevations above 1000 m are unfavorable for mosquito survival.

Another study reported that these mosquitoes can survive at altitudes lower than 700 m. This result aligns with findings in the present study, where the median elevation is 153.0 m for classic dengue fever and 201.0 m for hemorrhagic dengue, with survival recorded even at 1700 m [65]. Similarly, ref. [24] suggests that low-elevation areas favor dengue transmission. Additionally, ref. [66] confirmed that the flight ceiling of *Ae. aegypti* in Mexico from 1700 m to 2130 m. Ref. [67] detected *Ae. aegypti* vectors infected with dengue in Bellos, Colombia, at altitudes between 1900 and 2300 m. Conversely, it is stated that vectors inhabit below 1982 m [68]. Ref. [69] found a significant difference in mosquito percentages by elevation: 85.24% below 500 m, 13.06% below 1000 m, and 1.7% at 1000 m and above. These discrepancies in altitude parameters are attributed to mosquito adaptation and the interaction of environmental and population conditions. An inverse relationship was observed regarding the percentage of homes with piped water: as this percentage increased, vulnerability to dengue decreased. Without piped water, water must be stored in exposed containers, creating favorable conditions for mosquito breeding [70]. Similarly, the drainage index showed an inverse relationship: as the percentage of homes with drainage increased, vulnerability to dengue decreased.

Dengue not only spreads in clean water but also turbid water [71]. The methodology used in this study is relevant as decision-making in health has been supported by geographic information systems [72].

## 6. Conclusions

The findings can offer valuable insights for designing and implementing specific strategies by the State Health Services. It is also the responsibility of state and municipal authorities and society to address this complex issue holistically. The identified factors, such as population, the percentage of homes with piped water, and drainage index, can be modified to a certain extent. Since these factors are interrelated, altering one may simultaneously affect others.

Despite its contributions, this study has several limitations. Firstly, the analysis relies on aggregated data at the BGA level, which may mask fine-scale, household-level risk factors. Secondly, the model’s predictive power is constrained by the resolution and accuracy of the input data, such as the land use classification and climatic interpolations. Variables potentially related to the issue, such as schooling, education, and health promotion, were not explored. Furthermore, the cross-sectional nature of our data establishes associations but not causal relationships. Future research would benefit from longitudinal data and the inclusion of additional socioeconomic variables, such as mobility patterns and public health intervention data, to provide a more dynamic understanding of dengue vulnerability.

## Figures and Tables

**Table 1:** Variables used in the study.

Variable	Type	Parameter	Year	Source
**Dependent variables**				
Classic dengue fever	Health	Cases	2015–2021	[15]
Hemorrhagic dengue fever	Health	Cases	2015–2021	
**Independent variables**				
Elevation above sea level	Environmental	M	2020	[16,26]
Average annual temperature	Environmental	°C	2015–2020	[18]
Average annual rainfall	Environmental	Mm	2015–2020	
Average annual relative humidity	Environmental	%	2015–2020	[33]
Distance to bodies of water	Proximity	M	2015–2020	[25]
Distance to roads	Proximity	M	2015–2020	[26]
Distance to wooded areas	Proximity	M	2015–2020	[25]
Distance to grasslands	Proximity	M	2015–2020	[25]
Distance to agricultural areas	Proximity	M	2015–2020	[25]
XUTM	Location	M	2020	[26]
YUTM	Location	M	2020	[26]
Population	Social	Number of inhabitants	2015–2020	[30]
Homes with piped water	Social	%	2015–2020	[30]
Homes with drainage	Social	%	2015–2020	[30]

**Table 2:** Comparison of the means of the variables used in the study.

Variable ^¥^	Type of Dengue	Median	Mean	± SD	95% CI		Minimum	Maximum
					Upper Limit	Lower Limit		
Elevation above sea level (m) ^§ ℓ^	No dengue	0.0	610.7	785.1	583.4	638.1	0.0	2863.0
	Classic	153.0	529.2	627.5	510.1	548.2	10.0	2106.0
	Hemorrhagic	201.0	692.7	725.7	592.3	793.1	19.0	1943.0
Average annual temperature (°C) ^£^	No dengue	0.0	9.9	10.4	9.5	10.2	0.0	26.4
	Classic	24.2	22.9	2.5	22.8	23.0	16.8	27.1
	Hemorrhagic	23.7	22.2	2.9	21.8	22.6	17.2	2315.8
Average annual rainfall (mm) ^£^	No dengue	0.0	296.6	436.8	281.3	311.8	0.0	2.3
	Classic	1.5	1313.4	699.6	1292.2	1334.6	272.8	2393.5
	Hemorrhagic	1.5	1211.9	738.9	1109.6	1314.2	281.2	2265.6
Average annual relative humidity (%) ^£ℓ^	No dengue	65.3	65.5	6.6	65.3	65.7	52.9	77.8
	Classic	74.7	71.1	7.2	70.8	71.3	53.3	77.7
	Hemorrhagic	73.5	68.9	8.5	67.7	70.0	54.1	77.7
Distance to bodies of water (m) ^£^	No dengue	1.5	35,535.3	43,847.0	34,006.4	37,064.2	0.0	204,812.6
	Classic	1.5	2571.6	3042.8	2479.3	2663.9	0.0	25,497.1
	Hemorrhagic	1.9	2738.3	3457.5	2259.8	3216.7	0.0	19,440.4
Distance to roads (m) ^£^	No dengue	4.9	29,161.4	43,538.1	27,643.3	30,679.5	0.0	198,009.7
	Classic	90.0	170.4	218.9	163.8	177.1	0.0	2023.4
	Hemorrhagic	108.2	186.1	222.0	155.4	216.9	0.0	1171.5
Distance to grasslands (m) ^£^	No dengue	1.9	27,688.6	43,314.5	26,178.3	29,198.9	0.0	197,347.0
	Classic	60.0	127.2	269.9	119.1	135.4	0.0	2278.8
	Hemorrhagic	60.0	92.0	113.7	76.3	107.7	0.0	768.4
Distance to wooded areas (m) ^£^	No dengue	3.3	28,392.1	43,547.3	26,873.6	29,910.5	0.0	195,719.2
	Classic	123.7	411.5	1117.5	377.6	445.4	0.0	12,678.7
	Hemorrhagic	150.0	305.3	437.6	244.8	365.8	0.0	3390.9
Distance to agricultural areas ^£^	No dengue	5.0	30,742.9	45,429.5	29,158.8	32,326.9	0.0	200,867.4
	Classic	42.4	221.0	891.0	194.0	248.1	0.0	15,671.1
	Hemorrhagic	42.4	217.9	983.3	81.8	354.0	0.0	13,642.2
XUTM (m) ^£ ℓ^	No dengue	423,366.2	423,375.6	80,732.8	420,560.6	426,190.6	286,266.2	560,466.2
	Classic	502,266.2	466,630.2	80,774.7	464,180.8	469,079.6	281,676.2	565,356.2
	Hemorrhagic	498,726.2	445,355.8	93,106.9	432,470.5	458,241.0	288,456.2	565,626.2
YUTM (m) ^£ ℓ^	No dengue	2,509,896.2	2,509,887.0	98,151.3	2,506,464.6	2,513,309.4	2,342,631.2	26,77,131.2
	Classic	2,423,356.2	2,408,033.7	49,052.4	2,406,546.2	2,409,521.2	2,341,191.2	26,79,921.2
	Hemorrhagic	2,427,351.2	2,423,161.5	72,889.9	2,413,074.2	2,433,248.9	2,344,341.2	26,79,351.2
Population (*n*) ^£^	No dengue	128.1	304.8	3161.0	194.6	415.0	2.7	93,466.2
	Classic	620.8	7682.4	20,642.2	7056.5	8308.4	4.9	95,368.7
	Hemorrhagic	638.7	10,351.0	23,850.0	7050.3	13,651.6	7.3	92,941.6
Homes with piped water (%) ^£^	No dengue	16.0	24.1	23.2	23.3	24.9	0.0	96.8
	Classic	4.2	10.3	17.5	9.8	10.8	0.0	100.0
	Hemorrhagic	4.9	11.5	18.7	8.9	14.1	0.0	98.5
Homes with drainage (%) ^£^	No dengue	10.0	16.3	17.1	15.7	16.9	0.0	83.6
	Classic	8.0	12.5	13.8	12.1	13.0	0.0	90.1
	Hemorrhagic	9.1	13.5	14.2	11.5	15.4	0.0	63.9

^¥^ One-way OAOV. Significant difference in means (*p* < 0.05) between ^§^ no dengue and classic dengue fever; ^£^ no dengue with classic dengue fever and dengue hemorrhagic fever; ^ℓ^ classic dengue and dengue hemorrhagic fevers.

**Table 3:** Bivariate relationship between dengue cases and each independent variable. A significant relationship was indicated (*p* < 0.05). The beta coefficients are given in natural logarithm.

Variable	Classic Dengue Fever		Dengue Hemorrhagic Fever	
	Beta	*p*	Beta	*p*
Elevation above sea level	−0.165 × 10^−3^	<0.001	0.130 × 10^−3^	0.148
Average temperature	0.338	<0.001	0.258	<0.001
Average annual rainfall	0.268 × 10^−2^	<0.001	0.217 × 10^−2^	<0.001
Average annual relative humidity	0.104	<0.001	0.719 × 10^−1^	<0.001
Distance to bodies of water	−0.292 × 10^−3^	<0.001	−0.318 × 10^−3^	<0.001
Distance to roads	−0.405 × 10^−2^	<0.001	−0.436 × 10^−2^	<0.001
Distance to grasslands	−0.107 × 10^−2^	<0.001	−0.180 × 10^−2^	<0.001
Distance to wooded areas	−0.336 × 10^−3^	<0.001	−1.888	<0.001
Distance to agricultural areas	−0.287 × 10^−3^	<0.001	−0.529 × 10^−3^	<0.001
XUTM	0.634 × 10^−5^	<0.001	0.336 × 10^−5^	<0.001
YUTM	−0.176 × 10^−4^	<0.001	−0.192 × 10^−4^	<0.001
Population	0.767 × 10^−3^	<0.001	0.980 × 10^−4^	<0.001
Homes with piped water	−0.348 × 10^−1^	<0.001	−0.359 × 10^−1^	<0.001
Homes with drainage	−0.159 × 10^−1^	<0.001	−0.111 × 10^−1^	0.021

**Table 4:** Preliminary multivariate logistic models to estimate vulnerability to dengue fever. A significant relationship was indicated (*p* < 0.05). VIF > 10 indicates collinearity. The beta coefficients are given in natural logarithm.

Variable	Classic Dengue Fever				Dengue Hemorrhagic Fever			
	Beta	*p*	Tolerance	VIF	Beta	*p*	Tolerance	VIF
Elevation above sea level	−0.265 × 10^−2^	<0.001	0.163	6.134	−0.297 × 10^−2^	<0.001	0.081	12.346
Average temperature	0.198	<0.001	0.118	8.455	0.128	0.268	0.162	6.176
Average annual rainfall	0.911 × 10^−3^	<0.001	0.143	7.005	0.152 × 10^−2^	<0.001	0.147	6.813
Average annual relative humidity	−0.131	0.001	0.134	7.463	−0.369	0.001	0.547	1.830
Distance to bodies of water	−0.413 × 10^−4^	0.001	0.011	88.387	−0.837 × 10^−4^	0.008	0.009	107.735
Distance to roads	−0.389 × 10^−2^	<0.001	0.001	1000	−0.377 × 10^−2^	<0.001	0.001	1000
Distance to grasslands	0.975 × 10^−3^	<0.001	0.001	1000	−0.457 × 10^−4^	0.937	0.001	1000
Distance to wooded areas	0.143 × 10^−3^	0.024	0.002	500.000	−0.185 × 10^−3^	0.218	0.002	500
Distance to agricultural areas	−0.207 × 10^−4^	0.518	0.113	8.500	−0.971 × 10^−4^	0.272	0.015	66.470
XUTM	−0.174 × 10^−4^	<0.001	0.032	31.231	0.258 × 10^−5^	0.746	0.036	27.951
YUTM	−0.975 × 10^−5^	<0.001	0.323	3.099	0.379 × 10^−5^	0.083	0.041	24.390
Population	0.209 × 10^−3^	<0.001	0.698	1.433	0.706 × 10^−4^	<0.001	0.934	1.071
Homes with piped water	−0.676 × 10^−2^	0.006	0.639	1.566	−0.738 × 10^−2^	0.192	0.575	1.740
Homes with drainage	−0.111 × 10^−1^	0.003	0.667	1.499	−0.492 × 10^−2^	0.560	0.044	22.504
Constant	39.639	<0.001	--		13.040	0.057	--	

**Table 5:** Logistic models with standardized variables for estimating vulnerability to dengue fever. Significant relationship (*p* < 0.05); The beta coefficients are given in natural logarithm.

Variable	Classic Dengue Fever		Dengue Hemorrhagic Fever	
	Beta	*p*	Beta	*p*
Elevation above sea level	−2.365	<0.001	-	-
Average annual temperature	4.240	<0.001	4.029	0.017
Average annual rainfall	0.646	<0.001	1.936	<0.001
Annual relative humidity	−3.034	<0.001	−1.573	0.002
Distance to agricultural areas	−3.861	<0.001	-	-
YUTM	−0.894	<0.001	-	
Population	5.600	<0.001	1.169	<0.001
Homes with piped water (%)	−0.152	0.001	−0.384	<0.001
Homes with drainage (%)	−0.130	0.006	-	-
Constant	−0.674	0.039	−3.943	<0.001

**Table 6:** Percentage of the population vulnerable to dengue fever.

Level of Risk	Percentage of Population	
	Classic Dengue Fever	Dengue Hemorrhagic Fever
Very low	30.5%	92.0%
Under	6.1%	4.2%
Medium	8.0%	1.5%
High	9.2%	1.4%
Very high	46.3%	0.9%

## Data Availability

The original contributions presented in this study are included in the article. Further inquiries can be directed to the corresponding author.

## Abbreviations

IMTA	Mexican Institute of Water Technology
SMN	National Meteorological System
USGS	United States Geological Survey
BGA	Basic Geostatistical Area
UTM	Universal Transverse Mercator
INEGI	National Institute of Statistics and Geography
WGS84	World Geodetic System 1984
XUTM	Coordinate horizontal
YUTM	Coordinate vertical
%	Percentage
M or m	Meters
°C	Degrees Celsius
Mn	Millimeters
Z	Standardized unit
OAOV	One-Way Analysis of Variance
VIF	Variance Inflation Factor
¥	OAOV
§	Significant difference in means between no dengue and classic dengue fever
£	Significant difference in means between no dengue with classic dengue fever and dengue hemorrhagic fever
ℓ	Significant difference in means between classic dengue and dengue hemorrhagic fevers
AUC	Area under the curve
ROC	Receiver Operating Characteristic
